# Using non-invasive bi-level positive airway pressure ventilator via tracheostomy in children with congenital central hypoventilation syndrome: two case reports

**DOI:** 10.1186/s13256-015-0631-7

**Published:** 2015-06-25

**Authors:** Aroonwan Preutthipan, Teeradej Kuptanon, Harutai Kamalaporn, Anchalee Leejakpai, Malinee Nugboon, Duangrurdee Wattanasirichaigoon

**Affiliations:** 1 Division of Pediatric Pulmonology, Ramathibodi Hospital Sleep Disorder Center, Department of Pediatrics, Faculty of Medicine, Ramathibodi Hospital, Mahidol University, Payathai, Bangkok 10400 Thailand; 2 Department of Nursing, Faculty of Medicine, Ramathibodi Hospital, Mahidol University, Bangkok, 10400 Thailand; 3 Division of Medical Genetics, Department of Pediatrics, Faculty of Medicine, Ramathibodi Hospital, Mahidol University, Payathai, Bangkok 10400 Thailand

**Keywords:** Bi-level positive pressure ventilator, BPAP, CCHS, Children, Congenital central hypoventilation syndrome, Home mechanical ventilator, Long-term positive pressure ventilator, Tracheostomy

## Abstract

**Introduction:**

Due to the economic downturn in Thailand, two baby girls with congenital central hypoventilation syndrome had to wait for several months to obtain definite diagnosis and long-term mechanical ventilation. Genetic investigation later revealed 20/25 polyalanine expansion of *PHOX2B* gene in both girls. In this report we highlight the use of non-invasive bi-level positive airway pressure ventilators via tracheostomy, overnight end-tidal carbon dioxide trend graphs and outcomes of the patients whose diagnosis and treatment were delayed.

**Case presentation:**

Case 1: A Thai baby girl showed symptoms of apnea and cyanosis from birth and required invasive mechanical ventilation via tracheostomy during sleep. At 5 months, she unfortunately was discharged from the hospital without any ventilatory support due to financial problems. She subsequently developed cor pulmonale, respiratory failure and generalized edema and was referred to us when she was 9-months old. An overnight polysomnogram was consistent with a central hypoventilation disorder, in which the severity of oxygen desaturation and hypercapnia was worsening during non-rapid eye movement compared to rapid eye movement sleep. At 12 months she was allowed to go home with a conventional home ventilator. The ventilator was changed to bi-level positive airway pressure when she was 4-years old. After she received adequate home ventilation, she thrived with normal growth and development.

Case 2: A Thai baby girl developed apnea and cyanosis from the age of 5 weeks, requiring ventilatory support (on and off) for 5 months. After being extubated, she had been put on supplemental oxygen via nasal cannula for 2 months. She was then referred to us when she was 7-months old. An overnight end-tidal carbon dioxide trend graph revealed marked hypercapnia without increase in respiratory rate. An overnight polysomnogram was consistent with a central hypoventilation disorder. Since 9 months of age she has been on home bi-level positive airway pressure via tracheostomy without any complications.

Genetic testing confirmed 20/25 polyalanine expansions of *PHOX2B* gene in both girls.

**Conclusions:**

Bi-level positive airway pressure, originally designed as a non-invasive ventilator, was found to work effectively and safely, and may be used as an invasive ventilator via tracheostomy in young children with congenital central hypoventilation syndrome.

## Introduction

Congenital central hypoventilation syndrome (CCHS) is a rare disorder most commonly found in young children [1], characterized by alveolar hypoventilation and autonomic dysregulation [1, 2]. A mutation in the *PHOX2B* gene is requisite to establish the diagnosis [2, 3]. Since all patients do not outgrow the disorder, lifetime mechanical ventilation is the main treatment [2]. The morbidity and mortality outcomes may be variable depending on the adequacy of ventilatory support [2, 4].

The levels of financial support for homecare for children on long-term mechanical ventilation are different across the world. In high income countries such as in North America and Europe, ventilators, accessories and home healthcare professionals are generously provided by either a national health system or medical insurance [5–7]. However, in developing countries with limited resources it is much more difficult to send children with CCHS back home since all expense must be borne by the family themselves [8].

Our case studies feature two babies from a low socioeconomic background clinically diagnosed with CCHS. They eventually received home ventilators by donation. Genetic testing later confirmed *PHOX2B* mutation with 20/25 polyalanine expansion. In this report we highlight the use of bi-level positive airway pressure (BPAP) ventilators via tracheostomy, clinical manifestations and complications when home mechanical ventilation could not be initiated, our diagnostic approach when genetic tests were not available and outcomes after the patients received adequate ventilation.

## Case presentation

### Case 1

A Thai baby girl was born by caesarian section due to polyhydramnios, to a 38-year-old G_1_P_0_ mother, gestational age 38 weeks, birth weight 2500g. The baby was noted to have apnea and cyanosis since birth. At 10-days old, she was intubated due to frequent cyanosis and drowsiness. Six attempts of extubation failed due to apnea, hypopnea, cyanosis and hypercapnia. Tracheostomy was performed at 3 months of age. Ventilatory support was weaned to only at night time.

Due to financial problems, at 5-months old she unfortunately was discharged from the hospital without any ventilatory support. At home, during sleep she was noted to have intermittent cyanosis which occurred more often and worsened as time went by. The only thing her mother could do was to give tactile stimulation in order to wake her up.

At 9-months old, she developed cor pulmonale, respiratory failure, generalized edema and was referred to us in 2009.

On admission, a physical examination revealed body weight of 6.8kg (3rd percentile for age), height 67cm (25th percentile for age), head circumference 43cm, respiratory rate (RR) 35, and heart rate (HR) at 90 per minute. When staying awake her oxygen saturation measured by pulse oximetry (SpO_2_) was 95 to 98 % but when asleep SpO_2_ dropped to 58 to 60 %. Loud pulmonic valve closure (P_2_) and hepatomegaly were noted. Her hematocrit was 55 %. An electrocardiogram revealed right ventricular hypertrophy with strain pattern and right axis deviation.

Her past history was remarkable for recurrent aspiration pneumonia and generalized tonic–clonic seizures within the first month of life. Phenobarbital had been given to control seizures.

Clinical clues to diagnosis of CCHS included history of apnea, hypopnea, cyanosis and hypercapnia shortly after birth, and requirement of night time ventilatory support. The patient was able to breathe normally when awake. Four months after disconnection from the ventilator, she developed cor pulmonale most likely due to chronic hypoventilation during sleep. Other causes of hypoventilation including primary cardiac, pulmonary, metabolic and neuromuscular diseases were investigated and ruled out. At that time the genetic test for CCHS was not available in our country.

An overnight polysomnogram was performed, which revealed that when she was awake her RR was 42 per minute, SpO_2_ was 83 % in room air and increased to 98 % with supplemental oxygen (O_2_) 1 L per minute (LPM). During sleep her RR was 40 per minute. There were frequent central hypopneas of 30.4 events per hour. Peak end-tidal carbon dioxide (EtCO_2_) was 76mmHg which seemed to worsen during non-rapid eye movement (REM) compared to REM sleep. The duration that EtCO_2_ level was greater than 50mmHg was prolonged (43.5 % of total sleep time). Nadir SpO_2_ was 52 % with prolonged desaturation. There were no significant changes in RR and HR in response to increased carbon dioxide (CO_2_) and decreased SpO_2_.

Fortunately, a home ventilator (VS Integra™; ResMed, Sydney, Australia) was donated to her when she was 1-year old. Optimal setting of the ventilator from the titration under polysomnography was as followed: assisted pressure controlled mode, peak pressure 16, positive end-expiratory pressure (PEEP) 3cmH_2_O with back up rate 44 per minute. At this setting SpO_2_ was maintained at 96 to 98% with peak EtCO_2_ 47mmHg. No supplemental O_2_ was needed.

Her mother had been trained at bedside to give care for the tracheostomy, gastrostomy and ventilator for 1 month at our hospital. The patient returned home eventually and was put on a ventilator via tracheostomy only during sleep.

At the age of 4 years she grew to be healthy. Her weight, height and development were normal for a 4-year-old child. The first ventilator donated to her started to develop some problems 3 years after use. Fortunately, she received a second one also from a donation. During the 2-month wait for the second ventilator, her mother noted frequent cyanosis during sleep and more agitation during the daytime. Due to a limited budget, a bi-level device (VPAP III ST™; ResMed, Sydney, Australia) was selected. The setting was adjusted under polysomnography yearly. The ventilatory back up rate was decreased as she grew up.

At 5 years of age, her BPAP setting was adjusted to inspiratory pressure 17, expiratory pressure 5cmH_2_O, rate 20 per minute. She attended a regular school without any learning problems. Her weight and height were normal for her age. She demonstrated no clinical signs of cor pulmonale. Chest radiography and daytime arterial blood gases without ventilatory support were normal. An echocardiogram revealed improvement in pulmonary hypertension with estimated pulmonary artery pressure of 30 to 35mmHg.

### Case 2

A Thai baby girl was born by caesarean section to a 29-year-old G_1_P_0_ mother, gestational age 40 weeks, birth weight 4040g. She was found to have apnea and central cyanosis at 5-weeks old. SpO_2_ in room air was 80 %. She was then intubated and mechanically ventilated. An echocardiogram revealed dilated right atrium and right to left flow through foramen ovale. Extubation attempts repeatedly failed. After extubation, she could breathe spontaneously at the beginning; however, within 3 to 9 days she yet again developed respiratory failure which necessitated ventilatory support.

During sleep, she was noted to breathe slowly with RR of 18 to 20 per minute and she developed apnea and desaturation. Arterial blood gases on one occasion when she received only supplemental O_2_ at the previous hospital revealed pH 7.18, partial pressure of carbon dioxide in arterial blood (PaCO_2_) 76, partial pressure of oxygen in arterial blood (PaO_2_) 110mmHg and bicarbonate (HCO_3_) 28.4mEq/L. The peak PaCO_2_ was sometimes increased to 200mmHg. She had been intubated in the first and second hospitals on and off for 5 months.

CCHS was suspected but not confirmed. She had been extubated for 2 months at the second hospital before getting transferred to us when she was 7-months old in 2010. Oxygen 0.5LPM was given to her only during sleep.

**Fig. 1:**
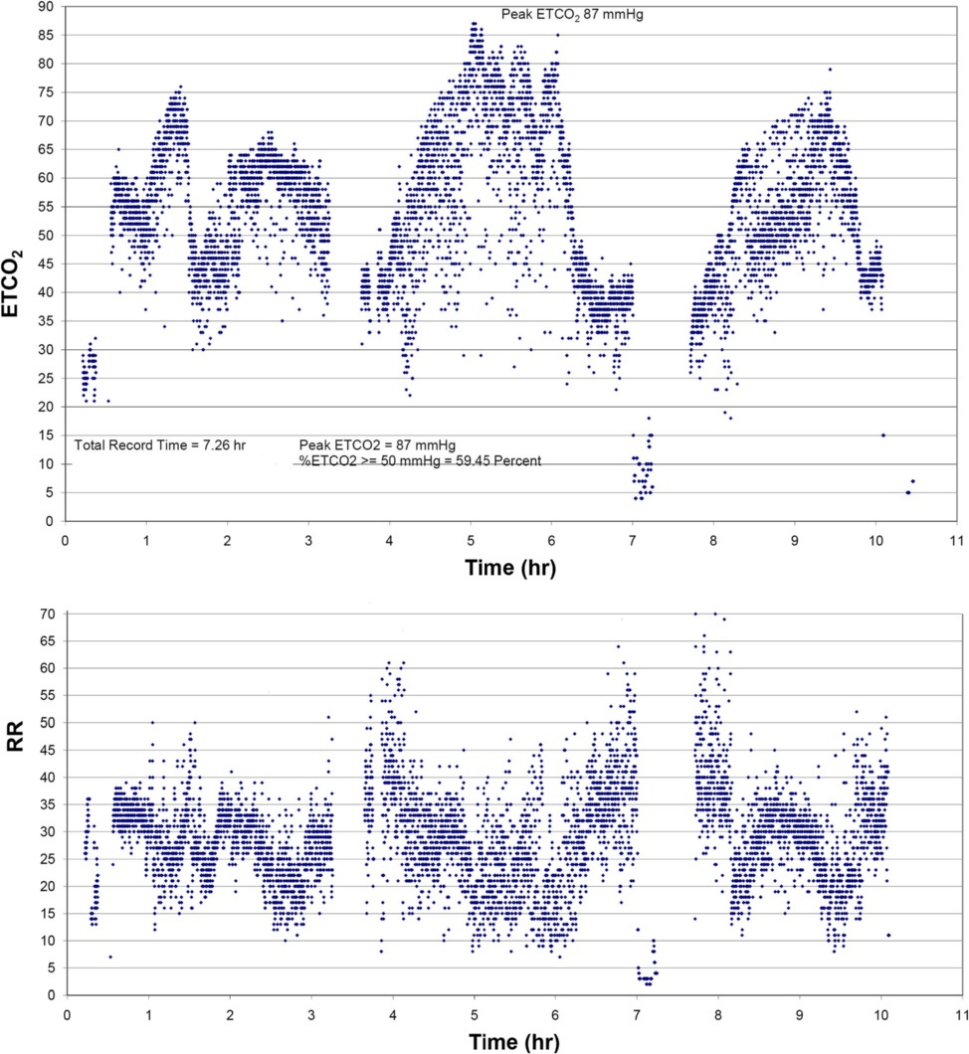
Overnight trend graphs of end-tidal carbon dioxide and respiratory rate. When end-tidal carbon dioxide increased to the peak level, respiratory rate did not increase indicating blunted physiologic response to hypercapnia. *ETCO*_*2*_ end-tidal carbon dioxide, *RR* respiratory rate

A physical examination on admission was unremarkable. Chest X-ray and echocardiogram were normal. She was put on O_2_ 0.5LPM during sleep. We recorded overnight EtCO_2_ and RR by using BCI Capnocheck®Plus (Smiths Medical, USA). Microsoft Excel program was used to create a trend graph of EtCO_2_ and RR monitoring. The EtCO_2_ was above 50mmHg during most of her sleep time and peak EtCO_2_ was elevated to 87mmHg. RR did not change in response to elevated EtCO_2_ (Fig. 1).

An overnight polysomnogram revealed that when she was awake, her RR was 40 and SpO_2_ was 96 to 98in room air. When she was asleep, her RR was decreased to 28 to 30 per minute. There were frequent central apneas of 2.8 events per hour and central hypopneas of 30.5 events per hour. The peak EtCO_2_ was 55mmHg. There was prolonged desaturation. Nadir SpO_2_ was 78 %. Hypercapnia was more prominent during non-REM sleep.

A tracheostomy was performed. We used a simple BPAP and titrated the setting under polysomnography. The most appropriate setting for her was inspiratory pressure 13, expiratory pressure 3cmH_2_O with back up rate of 35 per minute. SpO_2_ was maintained above 95 % with the peak EtCO_2_ of 50mmHg.

A BPAP ventilator (Airox Smartair ST™; Covidien, MA, USA) was donated to the patient. Her mother was trained by our nurses to be the caregiver. She had been on night time ventilatory support for more than 4 years without readmission. At 4 years of age, the BPAP setting was adjusted to inspiratory pressure 16, expiratory pressure 4cmH_2_O, rate 20 per minute. She has been doing well with normal growth and development.

Both patients were seen regularly at the pulmonary clinic. Polysomnographic studies were performed at least once a year to adjust ventilator settings that needed to change as the patients grew older.

### Genetic test

Genetic test using polymerase chain reaction (PCR) followed by direct sequencing of the exon 3 of the *PHOX2B* gene revealed 25 repetitions of polyalanine allele in addition to the normal allele with 20-alanine (GCG) residues, or designated as 20/25, in these two girls. The parents of both girls were found to carry only normal alleles. Primer sequences are as follows: forward 5′-CCAGGTCCCAATCCCAAC-3′ and reverse 5′-GAGCCCAGCCTTGTCCAG-3′.

## Discussion

We present the case of two girls with CCHS confirmed by *PHOX2B* mutation. Both have the same mutation of 25 repetitions of polyalanine (20/25 genotype), which is the most common mutation of the *PHOX2B* gene [2]. Our patients require ventilatory support only during sleep and did not have either Hirschsprung disease or tumors of neural crest origin or autonomic dysregulation. Without ventilatory support for 4 months, the first patient developed cor pulmonale, chronic respiratory failure, generalized edema and failure to thrive. She also had history of tonic–clonic seizures most likely due to persistent hypoxia.

The second patient did not demonstrate signs of cor pulmonale on admission to our hospital probably due to a less severe CCHS or earlier referral, and/or shorter period (only 2 months) of inadequate ventilation during sleep. She also had received night time supplemental O_2_ which could reduce hypoxia severity. We created overnight EtCO_2_ and RR monitoring graphs by using Microsoft Excel program. The graphs demonstrated a prolonged period of hypercapnia without any ventilatory response indicating abnormal physiologic control of ventilation. In a place where polysomnography and genetic testing are not available, an overnight EtCO_2_ and RR trend graph might be another simple method of screening for central hypoventilation.

As CCHS does not resolve spontaneously, nor does it appear to respond to pharmacologic stimulants or improve with advancing age, chronic ventilatory support at home has become a necessity that provides a chance for the patients to leave the hospital and lead a normal life at home with their family [2]. However in developing countries like Thailand, the cost of a home ventilator must be covered by the family themselves [8]. The first child was discharged from the hospital at 5 months of age without ventilatory support due to financial problems. Four months later she developed cor pulmonale, generalized edema and respiratory failure. She also had hemoconcentration as a compensation of prolonged hypoxemia. Without adequate ventilation in time the patient might have died. It should be noted that cor pulmonale was resolved after effective night time ventilatory support as shown in our patient.

**Fig. 2:**
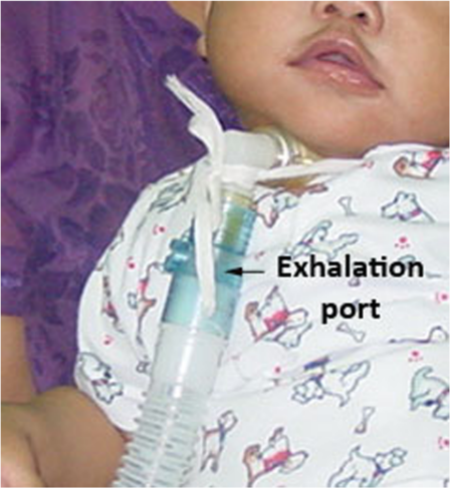
An exhalation port was connected proximal to the single limb circuit, closest to the tracheostomy

**Fig. 3:**
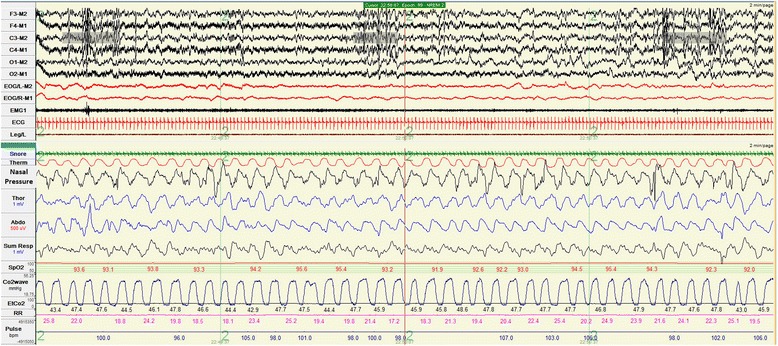
Polysomnographic tracings when the patient was breathing spontaneously. Desaturation and hypercapnia were present

**Fig. 4:**
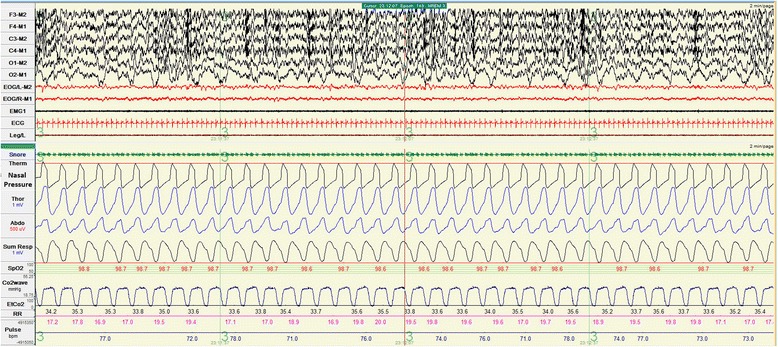
Polysomnographic tracing when the patient was put on bi-level positive airway pressure. There was neither oxygen desaturation nor hypercapnia. Nasal pressure tracing showed a pressure wave form generated by bi-level positive airway pressure

Because of the economic problems, we had to rely on donations to buy home mechanical ventilators for both patients. BPAPs originally designed as non-invasive ventilators were tried and used. A BPAP delivers a preset inspiratory positive airway pressure (IPAP) and expiratory positive airway pressure (EPAP). The tidal volume correlates with the difference between the IPAP and the EPAP. Back up rate was also set up in the timed mode of BPAP to guarantee breath delivery especially in small children who had relatively less effort to trigger the ventilator. Heated humidification systems were connected to the ventilator circuits. We attached an exhalation port in the single limb circuit closest to the tracheostomy in order to minimize rebreathing and prevent elevated CO_2_ (Fig. 2). In addition, the exhalation port does allow excessive inspiratory flow generated from BPAP to be released to the atmosphere. In terms of safety, the BPAP setting was to be titrated under polysomnography (Figs. 3 and 4). As our patients grew up, they required higher inspiratory pressure and lower back up rate. Up to the time of this report, no complications related to the use of BPAP were encountered. We demonstrated that BPAP via tracheostomy provided adequate ventilation and improved quality of life for young children with CCHS. More importantly its price is cheaper, approximately half of a conventional home ventilator. And in the future if the patients are older they may be able to switch to non-invasive ventilation via a nasal mask and use the old BPAP ventilator system [9] that they had got used to. In fact, our team at Ramathibodi Hospital, Bangkok, Thailand have been using home BPAP via tracheostomy in children with various problems since 1997. Up until now, 25 children have been put on long-term BPAP via tracheostomy. No complications have occurred so far [8].

Since home nursing care is impossible in our country, the mothers were trained to be caregivers [8]. Their education was only high school graduate or lower. They were trained at the bedside by our respiratory nurses on necessary components of respiratory care including tracheostomy care, O_2_ administration, suctioning, ventilator management, as well as basic life support. The patients were discharged after they were clinically stable with unchanged ventilator settings. The capabilities of the mothers to handle problems of children with mechanical ventilators were tested and approved by our team. Both children with CCHS who came from low income families have lived happily with normal growth and development. We showed that the mothers, who are not medical professionals, could provide safe ventilator care at home which results in improving the quality of life for the family and decreasing the cost of care, as compared to hospitalization. The members of both families were motivated and they dedicated their time and effort to their children.

## Conclusions

In conclusion, we reported the cases of two underprivileged girls diagnosed with CCHS. *PHOX2B* mutation of 25 repetitions of polyalanine was identified. BPAP ventilators were donated to them. The girls’ mothers were trained to be home caregivers. We found that BPAP ventilators were safe to be used via tracheostomy as long-term home mechanical ventilators in young children with CCHS.

## Consent

Written informed consents were obtained from the parents of both patients for publication of this case report and accompanying images. A copy of the written consents is available for review by the Editor-in-Chief of this journal.

## Abbreviations

BPAP: Bi-level positive airway pressure
CCHS: Congenital central hypoventilation syndrome
CO_2_: Carbon dioxide
EPAP: Expiratory positive airway pressure
EtCO_2_: End-tidal carbon dioxide
HR: Heart rate
IPAP: Inspiratory positive airway pressure
LPM: Liter per minute
O_2_: Oxygen
PaCO_2_: Partial pressure of carbon dioxide in arterial blood
REM: Rapid eye movement
RR: Respiratory rate
SpO_2_: Oxygen saturation measured by pulse oximetry
